# Dermatological and Dermoscopic Baselines in BRCA Mutation Carriers

**DOI:** 10.3389/fmed.2022.863468

**Published:** 2022-04-29

**Authors:** Giovanni Paolino, Riccardo Pampena, Matteo Riccardo Di Nicola, Caterina Longo, Alessia Rognone, Stefania Zambelli, Giampaolo Bianchini, Santo Raffaele Mercuri

**Affiliations:** ^1^Unit of Dermatology and Cosmetology, Istituto di Ricovero e Cura a Carattere Scientifico (IRCCS) San Raffaele Hospital, Milan, Italy; ^2^Azienda Unità Sanitaria Locale-Istituto di Ricovero e Cura a Carattere Scientifico (IRCCS) di Reggio Emilia, Centro Oncologico ad Alta Tecnologia Diagnostica-Dermatologia, Reggio Emilia, Italy; ^3^Department of Dermatology, University of Modena and Reggio Emilia, Modena, Italy; ^4^Department of Medical Oncology, Istituto di Ricovero e Cura a Carattere Scientifico (IRCCS) Ospedale San Raffaele, Milan, Italy

**Keywords:** BRCA1, BRCA2, mutations, dermatology, skin, angiomas, eruptive, dermoscopy

## Abstract

Breast cancer-associated genes 1 and 2 (BRCA1 and BRCA2) are tumor suppressor genes encoding a large protein that is involved in many essential biological processes. BRCA mutated patients show an increased risk to develop several malignancies, including cutaneous malignancies, although inconsistently across multiple studies. We carried out an observational study on the main dermatological and dermoscopic aspects in a population of patients with BRCA 1/2 mutations, to identify the main clinical and dermoscopical features in this class of patients. A total of 52 patients with BRCA mutations were included in the current analysis. Clinical, dermoscopical, and pathological data were obtained during the dermatologic visits. Out of the entire cohort, 67.3% of patients showed brown hairs and 63.5% of patients showed brown eyes, with phototype III as the most frequent phototype (69.2%). A total of 2.017 melanocytic lesions in all patients were analyzed; specifically, 40 patients (76.9%) showed a total number of nevi > 10, while regarding the main observed dermoscopic features, a prevalence of reticular pattern in 63% of cases was observed, followed by a mixed pattern in 19.2% of cases. Regarding the cutaneous examination, eruptive angiomas (eCAs) were the main dermatologic manifestations in 46.2% of patients. Out of 52 patients and during a follow-up of 24 months one patient developed an *in situ* melanoma. Interestingly, none of the patients with eCAs showed a TN > 10, highlighting an inverse correlation. To date, there is insufficient evidence to warrant increased surveillance in patients with BRCA mutations or with a positive family history for BRCA mutations, in the absence of standard cutaneous risk factors. Further studies with larger samples of patients are needed to better investigate dermatological and dermatoscopic features in BRCA mutation carriers.

## Introduction

Breast cancer-associated genes 1 and 2 (BRCA1 and BRCA2) are tumor suppressor genes encoding a large protein that is involved in many essential biological processes, including DNA damage repair, cell cycle checkpoints, chromatin remodeling, transcriptional regulation, and protein ubiquitination (1). Approximately 5% of breast cancers show this mutation. Women carrying BRCA mutations have a lifetime risk of developing breast cancer and/or ovarian cancer, of 45–75% and 18–40%, respectively (2). While, additional cancers reported in the BRCA1/2 spectrum include bone, oro-pharynx, esophagus, gallbladder and bile duct, laryngeal, ocular, male breast cancer, pancreas, stomach, and cutaneous malignancies, although inconsistently across multiple studies (3).

Research evaluating the association between BRCA1/2 mutations and skin cancers is limited; indeed, while no studies showed a statistically significant association between BRCA1 mutations and melanoma, the surveys investigating BRCA2 mutations and melanoma produced inconsistent conclusions (4). These inconsistent data were most likely caused by biases; indeed, in some studies, not all patients in the BRCA cohort were genetically tested (4); contrariwise, the studies that detectedthe occurrence of BRCA1/2 mutations in patients with a history of melanoma were mainly underpowered and demonstrated large variability in terms of genetic mutations and ethnic populations (4, 5). Therefore, the association between melanoma and non-melanoma skin cancers in patients with BRCA mutations remains an active area of investigation, and there are no official recommendations on to perform skin screening in BRCA1/2 mutation carriers.

In patients with BRCA mutations, structural changes may be already present in normal tissues, representing an increased risk of cancer (6). Furthermore, to our knowledge, there are no studies about the dermatological baselines in BRCA1/2 mutation carriers; herein, we carried out an observational study on the main dermatological features in a population of patients with an established BRCA1/2 mutation, to identify the main clinical and dermoscopic features in this class of patients.

## Materials and Methods

The study population (from 1 January 2019 to the end of October 2021) regarding a total of 16 BRCA1 and 36 BRCA2 germinal mutation carriers, referred to the IRCCS San Raffaele Hospital in Milano, were identified. Patients with a family history of breast and/or ovarian cancer were identified by clinicians from the Department of Oncology at the IRCCS San Raffaele Hospital in Milano. To be considered for BRCA1/BRCA2 mutation analysis, the family had to encompass at least 3 first degree relatives affected with breast or ovarian cancer (whereof at least one diagnosed before the age of 50 years), or 2 first degree relatives whereof at least 1 diagnosed before the age of 40 years. Patients with breast or ovarian cancer before the age of 30 were also considered suitable for the analysis. In addition, families with a case of male breast cancer were considered for BRCA1/2 mutation screening. The family members thus identified were classified as index patients.

All patients were referred to the Dermatology Unit at the IRCCS San Raffaele Hospital in Milano for cutaneous screening. For the evaluation of the cutaneous lesions, the manual dermoscope Heine Delta 20 T^®^ (HEINE Optotechnik GmbH & Co. KG Dornierstr. 6 82205 Gilching Germany) and the video-dermoscope Vidix 4.0^®^ (Medici Medical srl *Via* Panaro, 1, 41057 Spilamberto, Italy) were used.

**Figure 1:**
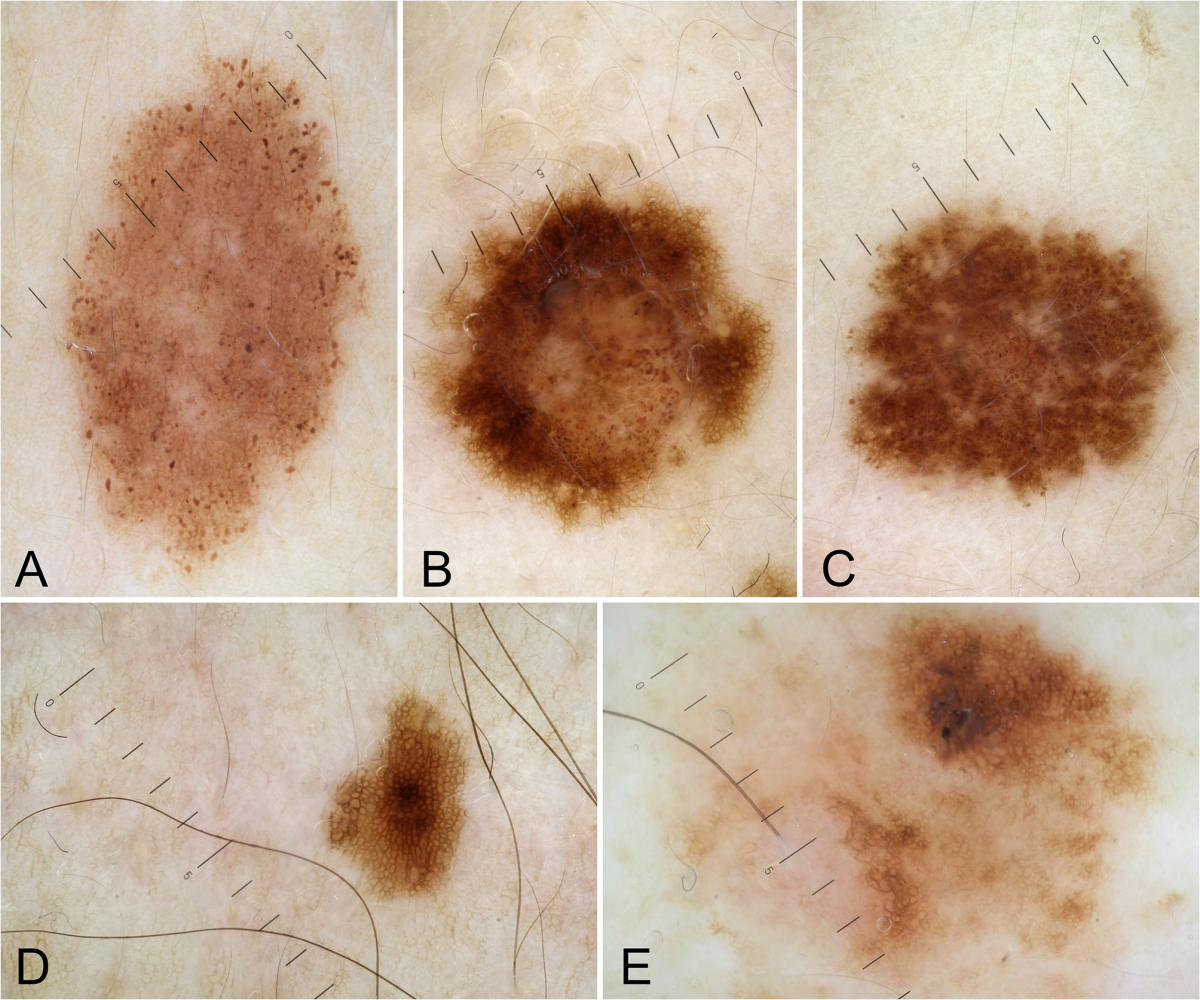
Dermoscopic patterns of nevi: uniform globular pattern **(A)**; mixed pattern composed of a central globular or structureless area surrounded by a network **(B)**; mixed pattern composed of a central network or structureless brown-gray area surrounded by a peripheral rim of small brown globules **(C)**; reticular pattern **(D)**; unspecified pattern **(E)**.

**Figure 2:**
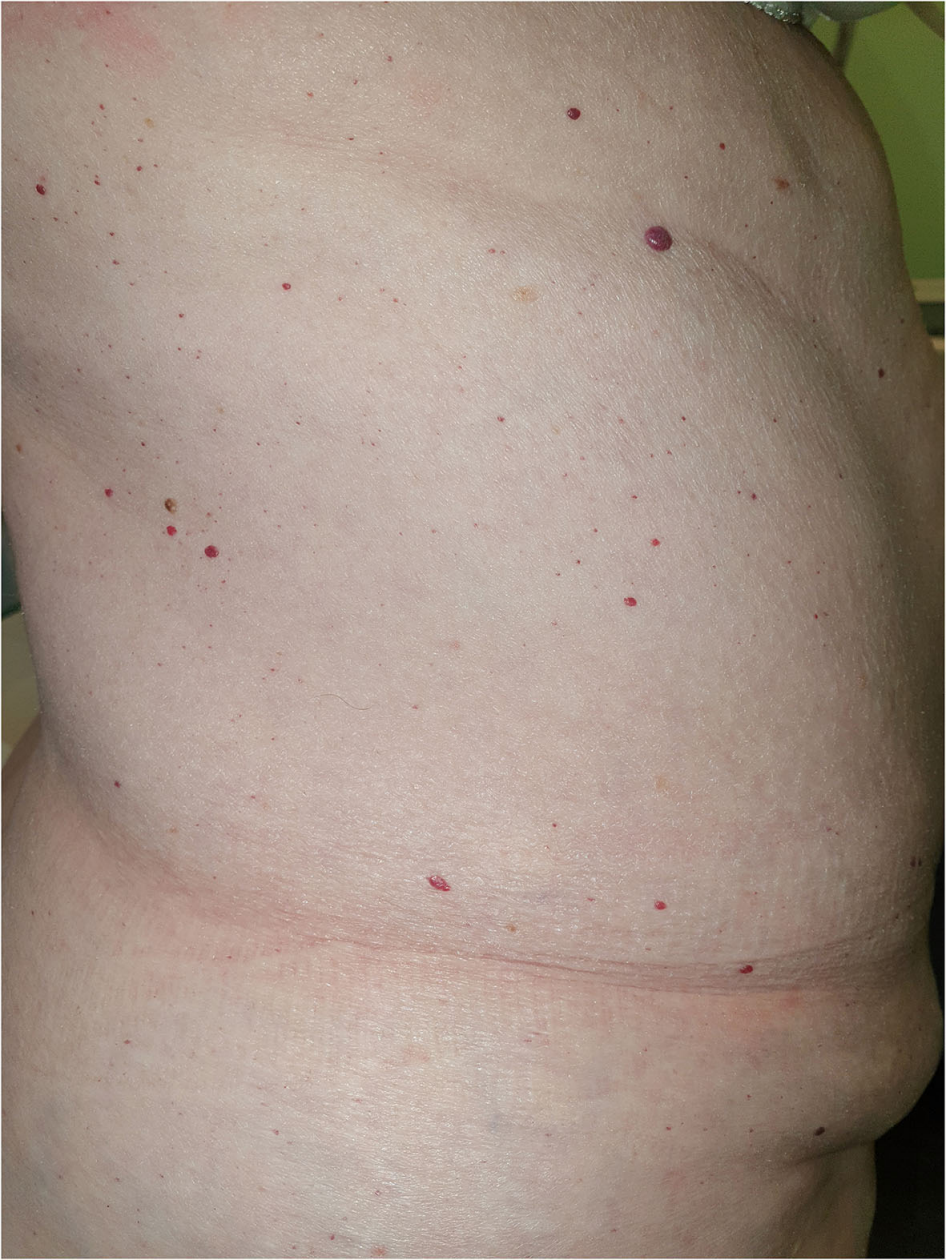
Eruptive cherry angiomas in a patient with breast cancer and BRCA1 mutation.

Clinical and pathological data were obtained during the dermatologic visits. The following parameters were registered as follows: age, gender, hair color, eye color, Fitzpatrick's phototype, personal and familial cancer history, personal and familiar cutaneous history, pigmented and non-pigmented dermatologic lesions detected during the visit, number and dermoscopic features of melanocytic lesions. Specifically, regarding melanocytic lesions, each nevus was dermoscopically classified according to a 5 pattern analysis, as reported in a previous article (7): uniform globular pattern, uniform reticular pattern, mixed pattern composed of a central globular or structureless area surrounded by a network, mixed pattern composed of a central network or structureless brown-gray area surrounded by a peripheral rim of small brown globules, and unspecified pattern, which encompassed all patterns other than the ones reported above (7) (Figures 1A-E). Besides, compared to Gandini et al. (8) we arbitrarily performed a count of total number of nevi (TN) for each patient, which was dichotomized into >10 and ≤10. While, in case of multiple cherry angiomas, they were classified as eruptive cherry angiomas (eCAs), when >10, as reported in two previous articles (9, 10) (Figure 2). Finally, given the high presence of patients with eCAs in our sample and according to recent studies (9, 10), we decided to study the subset of eCAs patients, considering also that angiogenesis has a very important role in malignancies and in BRCA mutations carriers.

## Results

### Demographics

The studied cohort consisted of 52 patients [5 men (9.6%), 47 women (90.4%)]; the median age of the whole group was 44 years (IQR 38–54.75 years). The median follow-up of the whole cohort was 24 months, ranging between 14 and 33 months. BRCA1 mutation was present in 16 patients (30.9%), while BRCA2 mutation was present in 36 patients (69.2%). Out of the entire cohort, 35 patients showed brown hair color (67.3%), 33 patients showed brown eyes (63.5%), while phototype III was the most frequent phototype (*n* = 36; 69.2%). A positive medical anamnesis for cancer was observed in 44.2% of cases (with breast cancer as the main observed cancer in 32.7% of cases), while the remaining 55.8% of patients did not show any positive personal medical history for cancer, performing periodic follow-up only for preventive purposes.

### Total Number of Nevi and Dermoscopic Analysis

A total of 2.017 melanocytic lesions were analyzed; specifically, 40 patients (76.9%) showed a TN > 10, while regarding the main observed dermoscopic features, the patients showed a prevalence of a reticular pattern in 63% of cases (*n* = 1.272), followed by a mixed pattern composed of a central globular or structureless area surrounded by a network in 19.2% of cases (*n* = 383), mixed pattern composed of a central network or structureless brown-gray area surrounded by a peripheral rim of small brown globules in 9.6% of cases (*n* = 193), uniform globular pattern in 5.7% (*n* = 115) and unspecified pattern in 2.7% of cases (*n* = 54).

### Angiomas

**Figure 3:**
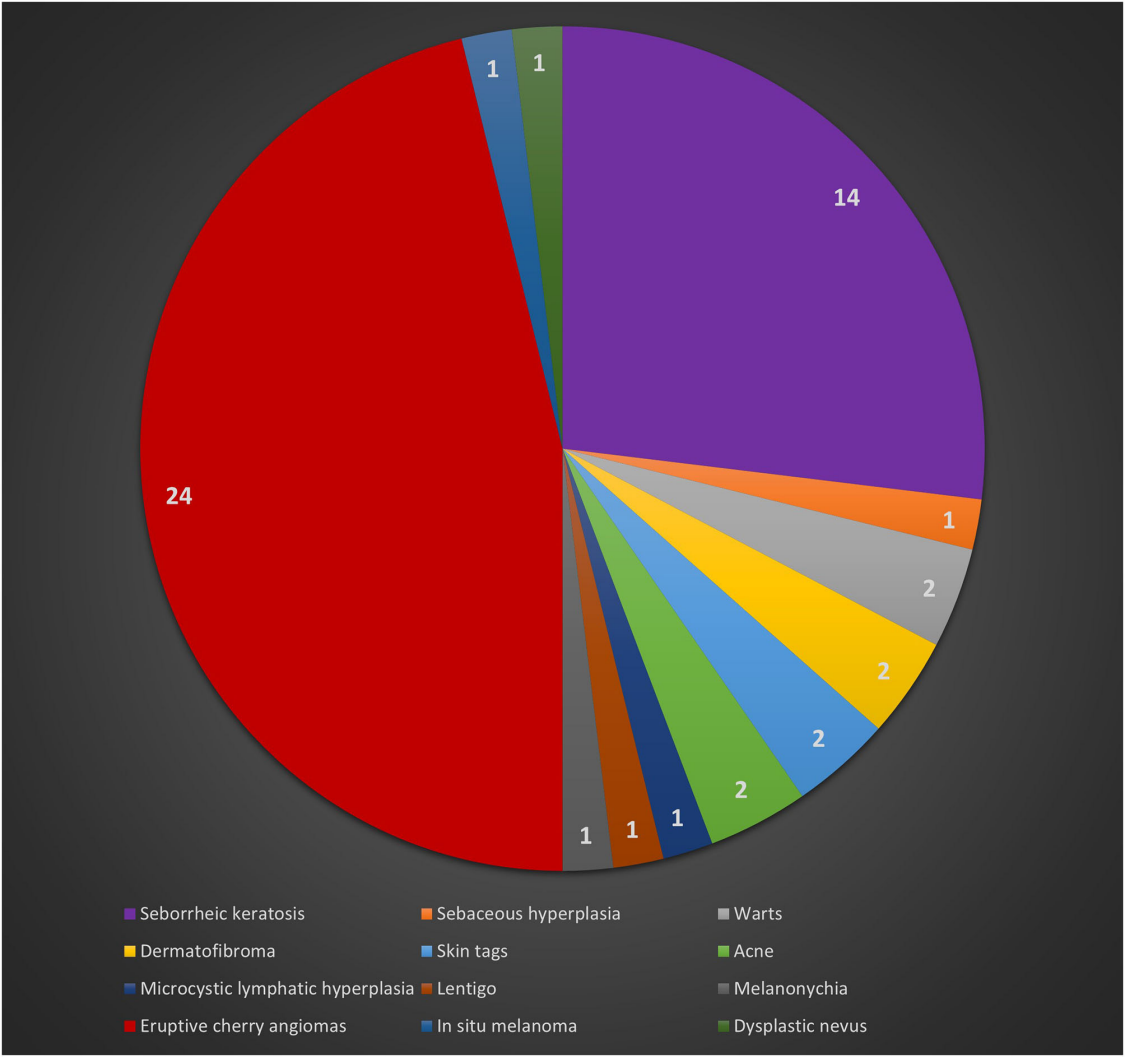
Occurrence of cutaneous lesions identified during the visits.

A negative dermatologic anamnesis was present in 37 cases (71.2%). Regarding the cutaneous examination, eruptive cherry angiomas (eCAs) were the main dermatologic manifestations in 24 patients (46.2%). Out of 52 patients and during a follow-up of 24 months, one patient developed an *in situ* melanoma. The remaining dermatologic manifestations are reported in Table 1 and Figure 3.

**Table 1:** Clinicopathologic features of the sample.

	***N***	**%**
**BRCA**		
BRCA1	16	30.8
BRCA2	36	69.2
**Gender**
Male	5	9.6
Female	47	90.4
**Hairs**		
Blond	9	17.3
Brown	35	67.3
Black	8	15.4
**Eyes**
Blue	15	28.8
Brown	33	63.5
Black	4	7.7
**FP**		
I	–	–
II	16	30.8
III	36	69.2
IV	–	
**PCH**
Negative	29	55.8
Positive^*^	26	44.2
**CA**		
Negative	37	71.2
Positive^**^	7	13.5
Unknown	6	11.5
**CL**
None	24	46.2
Positive	28	53.8
**eCAs**		
Negative	28	53.8
Positive	24	46.2
**TN**
>10	40	76.9
≤ 10	12	23.1
**Patterns of nevi** ^≠^		
R	1.272	63
MC	383	19.2
MP	193	9.6
G	115	5.7
U	54	2.7

*FP means Fitzpatrick's phototype*;

* *Positive personal cancer history (PCH): 18 breast cancer, 4 ovarian cancer, 1 prostate cancer*;

** *Positive cutaneous anamnesis (CA): actinic keratosis 1, basal cell carcinoma 1, atopic dermatitis 1, dermatofibroma 1, granuloma annulare 1, lipoma 1, prurigo 1, dysplastic nevus, unknown 6; cutaneous lesions identified during the visit CL: seborrheic keratosis 14, sebaceous hyperplasia 1, warts 2, dermatofibroma 2, skin tags 2, acne 2, microcystic lymphatic hyperplasia 1, lentigo 1, melanonychia 1, eruptive cherry angiomas 24, in situ melanoma 1, dysplastic nevus 1; TN means total number of nevi, with a mean number of nevi of 38 (standard deviation: 25.7). Regarding the patterns of nevi: uniform globular pattern (G), reticular pattern (R), mixed pattern composed of a central globular or structureless area surrounded by a network (MC), mixed pattern composed of a central network or structureless brown-gray area surrounded by a peripheral rim of small brown globules (MP), and unspecified pattern (U)*.

≠ *The analysis of the patterns of nevi was performed on a total of 2.020 melanocytic lesions that were evaluated in all 52 patients*.

Subsequently, we decided to evaluate a subset of patients with eCAs. They consisted of 2 men (8.3 %) and 22 women (91.7%); 8 were BRCA1 mutation carriers (33.3 %) and 16 were patients with a BRCA2 mutation (66.7%). Also, in this subset the main hair color was brown (*n* = 16; 66.7%), the main eye color was brown (*n* = 18; 75%), as well as the main phototype was III (*n* = 20; 83.3%). However, the percentage of patients with phototype III was higher in patients with eCAs vs. the ones without eCAs (83.3 vs. 16.7%; *p* = 0.04). The median age was 53.5 years (IQR 44-60 years) in eCAs vs. 41 (IQR 36.25-45 years) in patients without eCAs (*p* = 0.001). Interestingly, none of the patients with eCAs showed a TN > 10, with a median number of nevi of 3 (IQR 1-5) in patients with eCAs vs. 49 (IQR 22.5-67.5) in patients without eCAs (*p* = 0.04). Finally, regarding the main dermoscopic features of melanocytic lesions, they were similar in both samples (Table 2).

**Table 2:** Analysis according to the presence or absence of eruptive cherry angiomas.

	**Non-eCASs** ***(n** **=** **28)***		**eCAs** ***(n** **=** **24)***		***P***	**tot**	
	***n***	**%**	***n***	**%**		***n***	**%**
**BRCA**							
BRCA1	8	28.6	8	33.3	NS	16	30.8
BRCA2	20	71.4	16	66.7		36	69.2
**Gender**							
Male	3	10.7	2	8.3	NS	5	9.6
Female	25	89.3	22	91.7		47	90.4
Age	41	(36.2-45)	53.5	(44-60)	*0.001*	44	(38-54.7)
**Hairs**							
Blond	6	21.4	3	12.5	NS	9	17.3
Brown	19	67.9	16	66.7		35	67.3
Black	3	10.7	5	20.8		8	15.4
**Eyes**							
Blue	10	35.7	5	20.8	NS	15	28.8
Brown	15	53.6	18	75		33	63.5
Black	3	10.7	1	4.2		3	7.7
**FP**							
I	–	–	–	–	*0.04*	–	–
II	12	42.9	4	16.7		16	30.8
III	16	57.1	20	83.3		36	69.2
IV	–	–	–	–		–	
**PCH**							
Negative	16	57.1	13	54.2	NS	29	55.8
Positive^*^	12	42.9	11	45.8		23	44.2
**TN**							
> 10	23	82.1	0	–	*0.04*	23	44.3
≤ 10	5	17.9	24	100		29	55.7

*FP means Fitzpatrick's phototype*;

* *PCH means positive personal cancer history; TN means total number of nevi; NS means not statistically significant; In Italic statistically significant values*.

## Discussion

BRCA1 and BRCA2 are tumor suppressor genes that safeguard the genome (11) and cells lacking BRCA1 and/or BRCA2 are unable to repair double-stranded DNA breaks (4). Indeed, when cells are BRCA1/2 mutant, they are unable to perform homologous recombination, and DNA repair is pushed toward more error-prone pathways (4). Consequently, BRCA1/2 mutant cells may gain additional genetic mutations during DNA repair and can develop chromosomal aberrations during cellular replication (4). Many of these genetic mutations result in cell death, though some mutant daughter cells may survive and develop into a cell clone with malignant potential (4). These aberrations, not being confined only to the organs mainly known to be most affected by tumor processes (e.g., breast and ovary), may involve also other anatomic areas, where they allow the onset of possible tumors. In this regard, the skin (being the largest organ in the human body) may incorporate different mutations, giving rise to possible tumoral lesions.

Our sample consisted of 52 patients with BRCA1/2 mutations. Considering that in Italy the general incidence of BRCA mutations in patients with breast cancer is about 2.9% (12), the analysis of our case study is justified. In our sample, we had a higher percentage of BRCA2 mutation carriers; this result is interesting, since usually, BRCA1 shows a higher incidence compared to BRCA2, with a lifetime risk of developing breast cancer of 65% for BRCA1 mutation carriers and of 40% in patients with BRCA 2 mutation (13). BRCA2 is localized on chromosome 13q12, sharing structural and functional similarities with BRCA1 and codes for a 3,418 amino acid protein, which does not have structural analogies with BRCA1 protein (14). Besides, BRCA2 has a role in transcription regulation interacting with RAD51 and with p53 (14). Both RAD51 and p53 are known to be associated with several cutaneous diseases and malignancies (15, 16).

The most represented phototype in our population was phototype III (69.2%). We also detected brown hair (67.3%) and brown eyes (63.5%) as the most represented phototypes, followed by phototype II (30.8%). The predominance of phototype III can be explained by the high predominance of brown hairs and brown eyes among Italian people. Accordingly, the phototype of BRCA mutation carriers reflects the phototype mainly present in the specific population where the study was performed. Specifically, the phototype of BRCA mutation carriers is fairly normal, in contrast to MCR1R or CDKN2A patients, which contrariwise, are usually known to have red hair or multiple dysplastic nevi, respectively.

Regarding melanocytic lesions, the age-related prevalence of dermoscopic patterns in acquired melanocytic nevi (reticular as the main prevalent pattern) and a prevalence of TN > 10, reflected the ones of the general population (17).

Regarding the incidence of cutaneous malignancies, we diagnosed an *in situ* melanoma in only one patient with a BRCA2 mutation, while another BRCA2 mutation carrier reported a positive familiar history for melanoma in his sister, that died due to melanoma, carryingBRCA2 mutation too. However, to date, these data cannot demonstrate an association between melanoma and BRCA mutations, and further confirmations are needed.

Interestingly, excluding melanocytic lesions, the main associated cutaneous manifestations in patients with BRCA mutations were eCAs. The etiology of eCAs was not been fully elucidated but chemicals, immunosuppression, chronic graft-vs.-host disease, lymphoproliferative diseases, human herpesvirus 8 infections, and downregulation of microRNA-424 have been considered as potential inducers or predisposing factors for the development of eCAs (10). Despite the prevalence of eCAs in our sample (46.2%) was similar to the one in the general population (range 41–48%) (10), the detection of eCAs as the main cutaneous disorder in BRCA mutation carriers, compared to other common cutaneous lesions, such as solar lentigo and seborrheic keratosis, (which show a general prevalence of 79% in people aged 26-50 years) (18), or dermatofibromas (3% of all dermatopathology laboratory specimens) (19), highlighted the occurrence of eCAs in patients with BRCA mutations. The onset of eCAs is typically in the 40s−50s age group and they tend to increase in number and size with time; this could explain the relatively high incidence of eCAs in our sample (9, 10). Angiogenesis is a central event in the growth of both eCAs and neoplasms, including the ones associated with BRCA mutations (20). Previous reports found vascular endothelial growth factor (VEGF) and basic fibroblast growth factor (bFGF) to play a primary role in angiogenesis in malignancies associated with BRCA mutations (as various angiogenic and remodeling factors in tumor neovascularization) and both VEGF and bFGF are also necessary for the proliferation of eCAs (21, 22). These etiopathogenetic aspects could justify the detection of eCAs in our population of patients with BRCA mutations. Finally, according to a previous article (10), also in BRCA mutation carriers we confirm a possible inverse association between eCAs and TN, as a lower TN count was found in patients with BRCA mutations and eCAs.

One limitation of this report is that we did not compare the frequency of the dermatologic alterations found in the study group to those of a control population matched for age and clinical characteristics. However, considering our overall results (Tables 1, 2) compared to the general population, (4, 17–19) it does justify our choice to have studied this specific population of patients. Finally, we acknowledge the small sample size, but BRCA is not a common genetic mutation and it might be hard to collect larger samples of BRCA mutation carriers in single-center studies.

## Conclusion

Our findings highlighted that patients with BRCA mutations have a phototype that reflects the main phototype of the place where the analysis is carried out (phototype II and III, which are the main phototypes in Italy); an *in situ* melanoma was detected in only 1 patient, while eCAs were the main dermatologic manifestations detected in our sample, with an inverse association between eCAs and TN. There are no established guidelines for skin screening in BRCA mutation carriers. To date, there is insufficient evidence to warrant increased surveillance in patients with a confirmed BRCA mutation or with a positive family history for BRCA mutations, in the absence of standard cutaneous risk factors. Further studies with larger samples of patients are needed to better investigate dermatological and dermatoscopic features in BRCA mutation carriers.

## Data Availability Statement

The original contributions presented in the study are included in the article/supplementary material, further inquiries can be directed to the corresponding author/s.

## Ethics Statement

Ethical review and approval was not required for the study on human participants in accordance with the local legislation and institutional requirements. The patients/participants provided their written informed consent to participate in this study. Written informed consent was obtained from the individual(s) for the publication of any potentially identifiable images or data included in this article.

## Author Contributions

GP contributed to the conception, data collection, design of the study, and wrote the first draft of the manuscript. GP, RP, and MRDN organized the database and performed the statistical analysis. RP, MRDN, CL, AR, SZ, GB, and SRM contributed to writing sections of the manuscript. All authors contributed to the manuscript revision and read and approved the submitted version.

## Conflict of Interest

The authors declare that the research was conducted in the absence of any commercial or financial relationships that could be construed as a potential conflict of interest.

## Publisher's Note

All claims expressed in this article are solely those of the authors and do not necessarily represent those of their affiliated organizations, or those of the publisher, the editors and the reviewers. Any product that may be evaluated in this article, or claim that may be made by its manufacturer, is not guaranteed or endorsed by the publisher.
